# Effects of Scout Direction, Off-Centering, and Scout Imaging Parameters on Radiation Dose Modulation in CT

**DOI:** 10.3390/tomography12010005

**Published:** 2026-01-01

**Authors:** Yusuke Inoue, Hiroyasu Itoh, Hirofumi Hata, Kei Kikuchi

**Affiliations:** 1 Department of Radiological Sciences, Faculty of Health Sciences, Komazawa University, Tokyo 154-8525, Japan; 2 Department of Radiology, Kitasato University Hospital, Sagamihara 252-0375, Japan; hiroyasu@kitasato-u.ac.jp (H.I.); kmri@kitasato-u.ac.jp (H.H.); k-kiku@kitasato-u.ac.jp (K.K.)

**Keywords:** radiation protection, computed tomography, automatic exposure control, scout imaging, patient positioning

## Abstract

Background: In computed tomography (CT), automatic exposure control (AEC) determines the tube current and thus the radiation dose based on scout images. We investigated CT dose modulation using two versions of CARE Dose 4D, Siemens AEC software. Methods: A cylindrical phantom and an anthropomorphic phantom with the upper extremities raised or down were imaged. The CT tube current was determined using two versions of CARE Dose 4D and different scout directions: the posteroanterior scout image alone (PA scout), the lateral scout image alone (Lat scout), and the combination of the PA and Lat scout images (PA + Lat scout). The new version is designed to utilize the Lat image solely for off-center correction when both PA and Lat images are available. Experiments were performed at various vertical positions and with various scout imaging parameters. Results: The influence of the scout direction on CT dose was demonstrated, with variations depending on the imaging object and software version. The CT dose determined with the PA scout varied according to vertical positioning, presumably due to changes in image magnification. Such effects were small with the Lat scout or PA + Lat scout. Decreasing the tube voltage or tube current in scout imaging affected CT dose modulation with the Lat scout but not with the PA scout. With the PA + Lat scout, the effects of scout parameters were evident using the previous version but minimal using the new version. Conclusions: Off-center correction in the new version functioned appropriately. Because the behavior of an AEC system is complicated, it is recommended to examine the characteristics of each AEC system under various imaging conditions.

## Simple Summary

In computed tomography (CT), automatic exposure control (AEC) typically determines radiation exposure based on scout images, which are projection images obtained for CT planning. This experimental study demonstrated that the CT radiation dose determined by AEC varied with the scout imaging direction (posteroanterior, lateral, or both), vertical positioning of the imaging object, and scout imaging parameters. The influences of these factors depended on the imaging object and the AEC software version. The off-center correction incorporated into the new version was demonstrated to function appropriately and to contribute to a reduction in radiation dose from scout imaging.

## 1. Introduction

Computed tomography (CT) delivers relatively high radiation doses to patients, raising concerns regarding increased risks of radiation-induced cancer [1,2,3,4]. Optimization of CT is a key issue in contemporary clinical medicine. To optimize radiological imaging, radiation doses are reduced while preserving image quality and diagnostic performance. In CT, a patient is exposed to X-rays, and transmitted photons are detected to reconstruct tomographic images. Higher radiation exposure is required in larger patients to preserve image quality, due to greater X-ray attenuation. Automatic exposure control (AEC) is widely used to achieve appropriate radiation dose modulation according to the degree of attenuation by the patient. In CT examinations, projection images, termed scout images or localizer images, are acquired prior to tomographic imaging to define the scan range. AEC software estimates attenuation by the patient primarily using the scout images to determine the tube current for tomographic imaging. The radiation output is proportional to the tube current. AEC enables radiation dose modulation taking into account differences in attenuation severity among patients and among regions within a patient and is accepted as an essential tool for optimization [5,6].

Radiation dose modulation determined by AEC is influenced by various factors, including the type of AEC software [7,8,9], AEC parameter settings [7,9], scout imaging direction [7,8,10,11,12,13], patient positioning [11,14,15,16], arm positioning [7,10,17], and scout imaging parameters [18,19]. Positioning the patient at the isocenter of the CT scanner is essential for appropriate AEC-based dose modulation. When posteroanterior (PA) and anteroposterior (AP) scout images are used for AEC, positioning the patient below and above the scanner isocenter, respectively, increases radiation exposure due to enhanced image magnification [11,14,15,16]. Previous studies have demonstrated that the effect of vertical off-centering on dose modulation is larger with a PA or AP scout image than with a lateral (Lat) scout image [14,15,16]. To avoid off-center positioning, automatic patient positioning methods have been developed [20]. CARE Dose 4D is the AEC system installed in the CT systems provided by Siemens. When both PA and Lat scout images are acquired, CARE Dose 4D determines radiation dose modulation using both images. In the new version of CARE Dose 4D, the CT tube current is first determined based on the patient attenuation estimated from the PA scout image, and the Lat scout image is used solely for off-center correction based on the patient’s vertical position.

In positron emission tomography (PET)/CT for oncology practice, whole-body CT is usually performed together with PET mainly for attenuation correction of PET images and localization of lesions detected by PET. CT offers valuable supplementary information but increases radiation dose substantially. The CT component of PET/CT delivers variable radiation doses and is an important target for optimization. Additionally, because scout images covering the whole body are acquired, optimization of scout imaging is desirable. Decreasing the tube voltage or tube current in scout imaging reduces the radiation dose derived from scout imaging itself; however, it may affect CT dose modulation [18,19]. A chest phantom study using the previous version of CARE Dose 4D demonstrated that decreasing the tube voltage or tube current in scout imaging reduced CT radiation exposure around the lung apex when dose modulation was performed based on the Lat scout image or the combination of the PA and Lat images [18]. The cross-section around the lung apex has a large right-to-left diameter and contains a large amount of bone, owing to the presence of the shoulders, leading to strong X-ray attenuation in the Lat direction. On the low-voltage, low-current Lat image, the strong X-ray attenuation was underestimated, and image contrast was low, which appeared to be responsible for the reduced CT radiation exposure. Similar to chest CT, decreasing the tube voltage or tube current may disturb accurate estimation of attenuation on the Lat image in whole-body PET/CT, affecting CT dose modulation using the previous version of CARE Dose 4D. The impact of the quality of the Lat scout image on CT dose modulation may be reduced when using the new version because only the vertical position is assessed on the Lat image.

In the present study, CT of cylindrical and anthropomorphic phantoms was performed using both the previous and new versions of CARE Dose 4D. We examined the effects of the scout imaging direction, vertical off-centering, and scout imaging parameters on CT radiation dose modulation. The primary aim of this study was to verify whether the off-center correction function in the new version works as intended and to assess its implications.

## 2. Materials and Methods

### 2.1. Instruments

A 64-detector-row CT scanner SOMATOM Definition Flash with Stellar detector (Flash; Siemens, Erlangen, Germany) and a PET/CT scanner Biograph mCT Flow (mCT; Siemens) were used. The mCT scanner consisted of a PET scanner and a 32-detector-row CT scanner. In the Flash scanner, the operating system was SOMARIS 7 VA48A, and the AEC system was the previous version of CARE Dose 4D. In the mCT scanner, the operating system was SOMARIS 7 VB20, and the AEC system was the new version of CARE Dose 4D.

Using these scanners, a 32 cm cylindrical phantom filled with water and an anthropomorphic whole-body phantom (PBU-60; Kyoto Kagaku, Kyoto, Japan) were imaged.

### 2.2. Effects of Vertical Positioning in Imaging the Cylindrical Phantom

The 32 cm cylindrical phantom was imaged at various vertical positions on the two scanners to examine the CT tube current determined by AEC. First, the phantom was positioned so that its center coincided with the isocenter of the scanner. To ensure accurate centering, after positioning the phantom using the laser alignment system, it was imaged with CT and repositioned if necessary. Thereafter, scout imaging was performed at the isocenter, followed by CT. The table height was then changed in 2 cm increments, and scout imaging followed by CT was performed at positions ranging from 6 cm below to 6 cm above the isocenter.

The tube current for CT was determined based on the PA scout image alone (PA scout), the Lat scout image alone (Lat scout), and the combination of the PA and Lat scout images (PA + Lat scout). The parameters for scout imaging were set according to the manufacturer’s recommendations as follows: tube voltage, 120 kV; tube current, 35 mA; beam width, 3.6 mm; and table speed, 100 mm/s (Flash) or 200 mm/s (mCT). CT was performed with the following settings: axial mode; tube voltage, 120 kV; rotation time, 1 s; beam width, 38.4 mm (Flash) or 19.2 mm (mCT); slice thickness/interval, 5/5 mm; scan field of view (FOV), 500 mm; and display FOV, 340 mm. For CARE Dose 4D, the quality reference mAs was set at 240 mAs, and the strength of tube current modulation was average decrease (slim) and average increase (obese) modulation.

The resulting tube current in CT was recorded, and its relationship with the vertical position was evaluated in relation to the version of CARE Dose 4D and the scout direction.

### 2.3. Effects of Vertical Positioning in Imaging the Anthropomorphic Phantom

The anthropomorphic phantom was imaged at various vertical positions on the two scanners to evaluate longitudinal CT dose modulation determined by AEC, simulating clinical thoraco-abdominopelvic CT. The upper and lower extremities were attached to the trunk of the phantom, with the upper extremities raised. The scan range for CT covered from the thorax to the pelvis, with a scan length of 65 cm. The phantom was initially positioned so that its center at the level of the first lumbar vertebra coincided with the scanner isocenter, and CT images were obtained after scout imaging. The imaging table was then moved 5 cm upward and 5 cm downward, and scout imaging followed by CT was performed at each position.

The CT tube current was determined based on the PA scout, Lat scout, and PA + Lat scout. The scout imaging parameters were the same as those used to image the cylindrical phantom. CT was performed in the helical mode and in the cranio-caudal direction. Other imaging parameters for CT were as follows: tube voltage, 120 kV; rotation time, 1 s; pitch, 0.8; beam width, 38.4 mm (Flash) or 19.2 mm (mCT); slice thickness/interval, 5/5 mm; scan FOV, 500 mm; and display FOV, 400 mm. For CARE Dose 4D, the quality reference mAs was set at 400 mAs, and the strength of tube current modulation was average decrease (slim) and average increase (obese) modulation.

The tube current for each slice position was extracted from the DICOM tags to generate dose modulation curves. The effect of vertical positioning on longitudinal CT dose modulation was evaluated in relation to the CARE Dose 4D version and the scout direction.

### 2.4. Effects of Scout Imaging Parameters

The anthropomorphic phantom was imaged using various tube voltages and tube currents for scout imaging on the two scanners to evaluate the effects of scout imaging parameters on CT dose modulation determined by AEC, simulating the CT component of whole-body oncology PET/CT. In whole-body PET/CT, the upper extremities may be positioned either raised or down. In this study, we simulated imaging with the upper extremities down: the upper extremities were placed just beside the trunk, and the lower extremities were attached to the trunk. The scan range for CT covered from the vertex to the proximal thigh, with a scan length of 95 cm. The phantom was positioned so that its center at the level of the xiphoid process coincided with the scanner isocenter, and CT images were obtained after scout imaging.

The CT tube current was determined using the PA scout, Lat scout, and PA + Lat scout. Scout imaging was performed using nine combinations of tube voltages (120, 100, and 80 kV) and tube currents (100, 35, and 20 mA). Other scout parameters were a beam width of 3.6 mm and a table speed of 100 mm/s (Flash) or 200 mm/s (mCT). CT was performed in the helical mode and in the cranio-caudal direction, with a rotation time of 0.5 s, a quality reference mAs of 180 mAs, and a display FOV of 500 mm. Other CT parameters were the same as those used in the experiments in which the anthropomorphic phantom was imaged at various vertical positions.

The tube current for each slice position was extracted from the DICOM tags to generate dose modulation curves. Additionally, the volume CT dose index (CTDIvol) calculated by the CT scanner was recorded and expressed as a percentage of the value obtained at 120 kV and 100 mA for each scanner to obtain relative CTDIvol. The effects of scout imaging parameters on CT dose modulation were evaluated.

## 3. Results

### 3.1. Effects of Vertical Positioning in Imaging the Cylindrical Phantom

**Figure 1:**
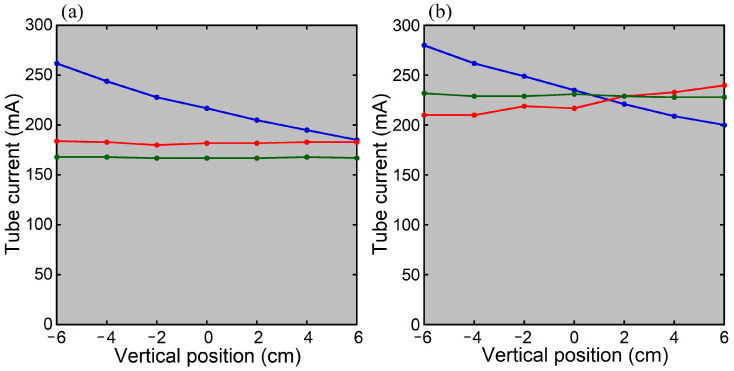
CT tube currents and vertical positions in imaging the cylindrical phantom using the previous (**a**) and new (**b**) versions of CARE Dose 4D. The blue, red, and green lines indicate the results with the PA scout, Lat scout, and PA + Lat scout, respectively.

When the cylindrical phantom was positioned at the isocenter of the scanner (vertical position = 0 cm), the tube current determined using the previous version of CARE Dose 4D was higher with the PA scout than with the Lat scout or PA + Lat scout (Figure 1). Using the new version, the tube current was slightly lower with the Lat scout than with the PA scout or PA + Lat scout, but the differences among the scout directions were small.

When the PA scout was used, the CT tube current decreased as the phantom was positioned higher, regardless of the CARE Dose 4D version (Figure 1). Using the previous version with the Lat scout or PA + Lat scout, the CT tube current remained constant regardless of vertical positioning. Using the new version, it increased slightly at higher positions with the Lat scout, whereas it remained constant with the PA + Lat scout.

### 3.2. Effects of Vertical Positioning in Imaging the Anthropomorphic Phantom

**Figure 2:**
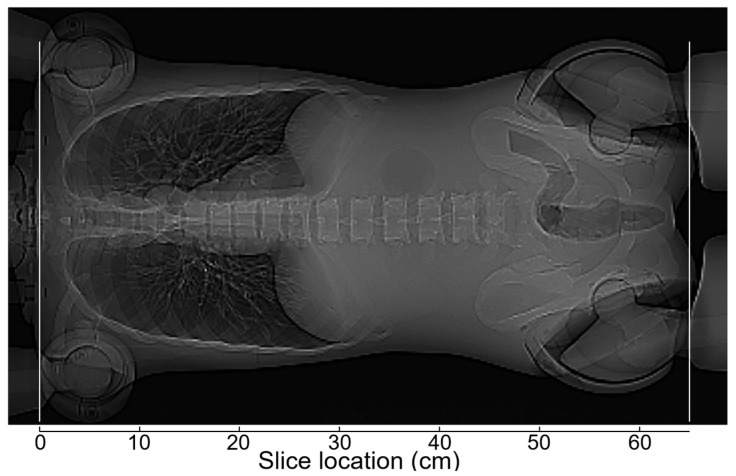
PA scout image obtained in the experiment assessing the effects of vertical positioning in imaging the anthropomorphic phantom. The phantom positioned at the isocenter was imaged on the mCT scanner, with the upper extremities raised. The vertical white lines indicate the upper and lower borders of the scan range.

**Figure 3:**
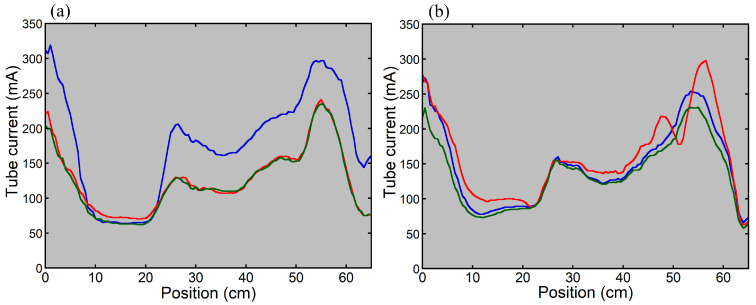
Tube current modulation curves for imaging the thoraco-abdominopelvic region of the anthropomorphic phantom using the previous (**a**) and new (**b**) versions of CARE Dose 4D. The phantom was positioned at the scanner isocenter. The blue, red, and green lines indicate the results with the PA scout, Lat scout, and PA + Lat scout, respectively.

Figure 2 shows a PA scout image in thoraco-abdominopelvic imaging of the anthropomorphic phantom with the upper extremities raised. When the phantom was positioned at the isocenter of the scanner, the dose modulation curve determined using the previous version of CARE Dose 4D was almost identical between the Lat scout and PA + Lat scout (Figure 3). With the PA scout, the CT tube current was higher than that with the Lat scout or PA + Lat scout, except in the mid-thoracic region. The influence of the scout direction on tube current modulation was smaller using the new version than using the previous version. With the Lat scout, the shape of the dose-modulation curve in the pelvic region differed from that obtained with the PA scout or PA + Lat scout. The PA scout and PA + Lat scout produced dose modulation curves with generally similar shapes.

**Figure 4:**
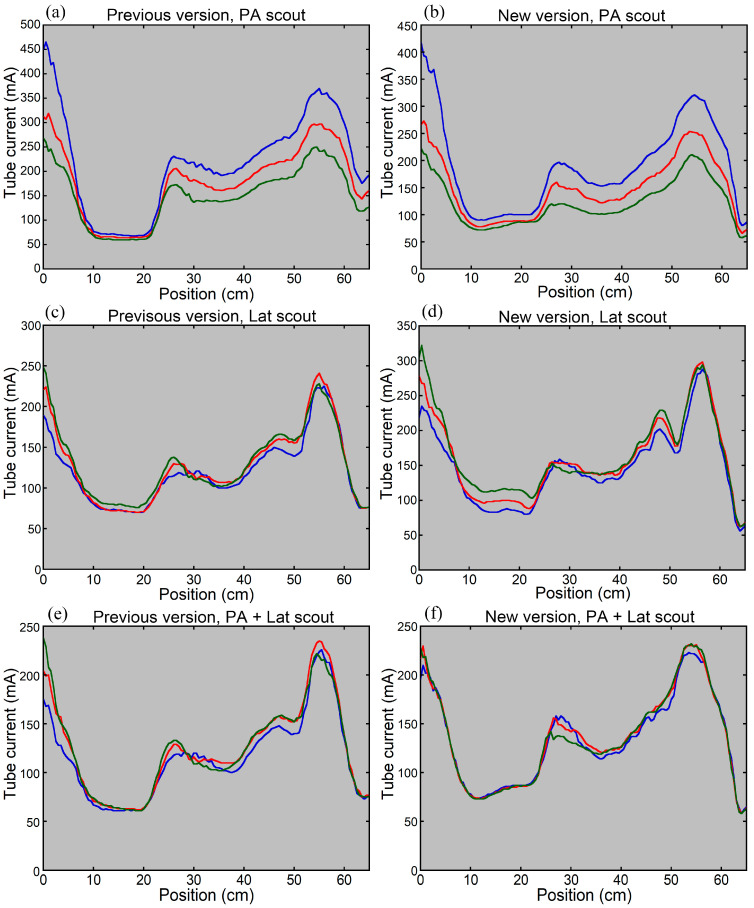
Tube current modulation curves for imaging the thoraco-abdominopelvic region of the anthropomorphic phantom using the previous (**a**,**c**,**e**) and new (**b**,**d**,**f**) versions of CARE Dose 4D. The CT tube current was determined based on the PA scout (**a**,**b**), Lat scout (**c**,**d**), or PA + Lat scout (**e**,**f**). The blue, red, and green lines indicate the results obtained for the phantom positioned 5 cm below the isocenter, at the isocenter, and 5 cm above the isocenter, respectively.

When the phantom was imaged at different vertical positions using the previous version of CARE Dose 4D, the CT tube current increased at the lower position and decreased at the higher position with the PA scout (Figure 4). Imaging the phantom at the lower position increased its lateral width on the obtained PA scout image; however, it remained within the scan range. The effect of vertical positioning on tube current modulation was smaller with the Lat scout or PA + Lat scout. When using the new version, the CT tube current increased at the lower position and decreased at the higher position with the PA scout. The influence of vertical positioning was reduced with the Lat scout and was almost eliminated with the PA + Lat scout.

### 3.3. Effects of Scout Imaging Parameters

**Figure 5:**
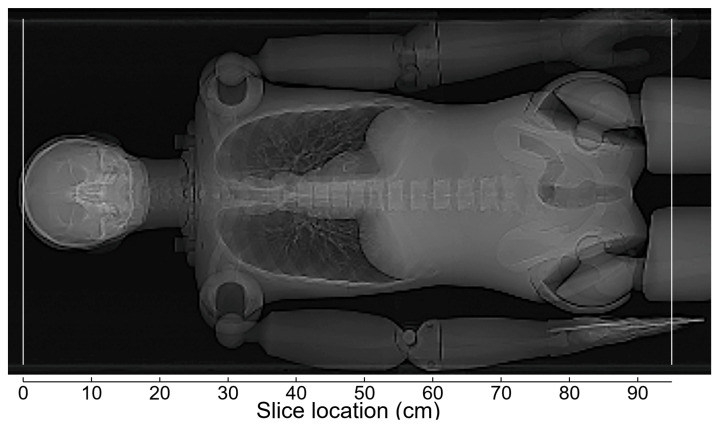
PA scout image obtained in the experiment assessing the effects of scout imaging parameters. The anthropomorphic phantom was imaged on the mCT scanner with the upper extremities beside the trunk. The vertical white lines indicate the upper and lower borders of the scan range.

**Figure 6:**
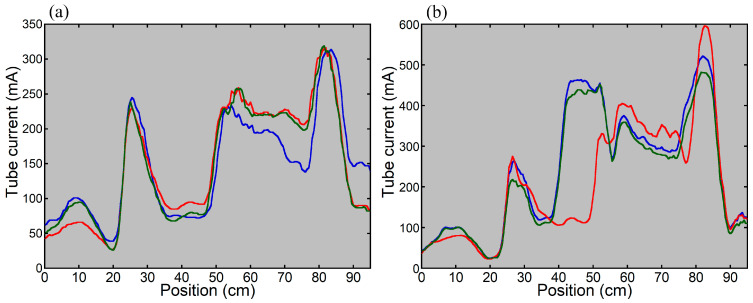
Tube current modulation curves for whole-body imaging of the anthropomorphic phantom using the previous (**a**) and new (**b**) versions of CARE Dose 4D. Scout imaging was performed at a tube voltage of 120 kV and a tube current of 100 mA. The blue, red, and green lines indicate the results with the PA scout, Lat scout, and PA + Lat scout, respectively.

Figure 5 shows a PA scout image in whole-body imaging of the anthropomorphic phantom with the upper extremities beside the trunk. When scout imaging was performed at a tube voltage of 120 kV and a tube current of 100 mA, the CT tube current determined using the previous version of CARE Dose 4D was higher in the head and proximal thigh and lower in the trunk with the PA scout than with the Lat scout (Figure 6). The dose modulation curve obtained with the PA + Lat scout was similar to that with the PA scout from the head to the chest and to that with the Lat scout in the abdominopelvic region. Using the new version, the dose modulation curves were generally similar between the PA scout and PA + Lat scout. The CT tube current determined with the Lat scout differed from that with the PA scout or PA + Lat scout, particularly in the lower thoracic region.

**Figure 7:**
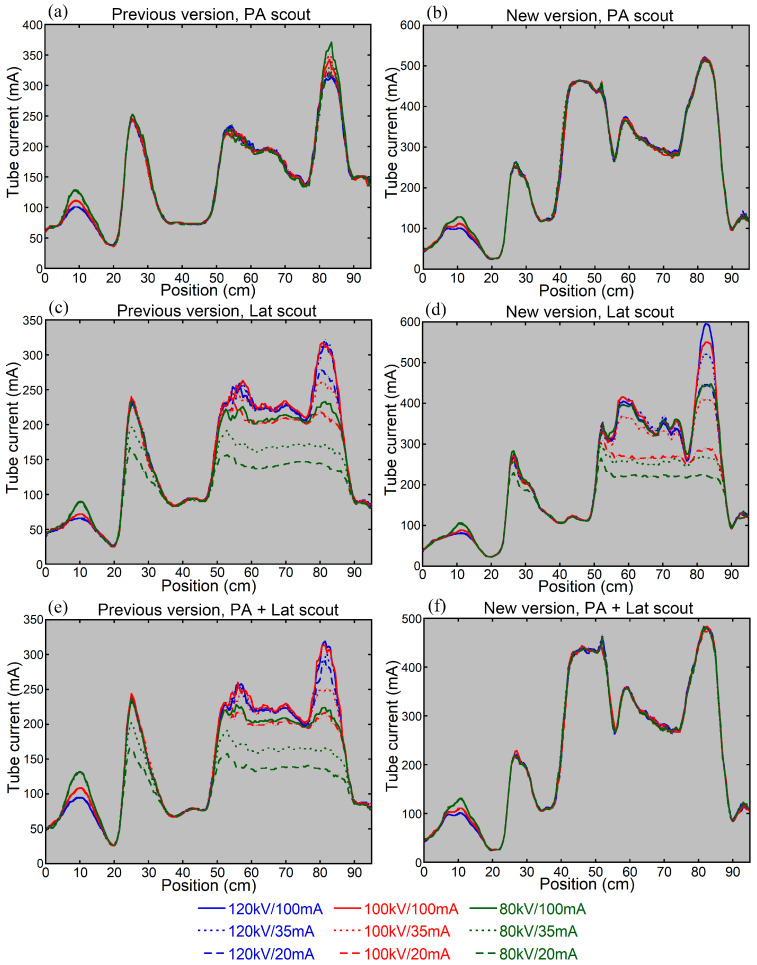
Tube current modulation curves for whole-body imaging of the anthropomorphic phantom using the previous (**a**,**c**,**e**) and new (**b**,**d**,**f**) versions of CARE Dose 4D. The CT tube current was determined based on the PA scout (**a**,**b**), Lat scout (**c**,**d**), or PA + Lat scout (**e**,**f**). The blue, red, and green lines indicate results derived from scout imaging at tube voltages of 120, 100, and 80 kV, respectively, and the solid, dotted, and dashed lines indicate results at tube currents of 100, 35, and 20 mA, respectively.

When the CT tube current was determined using the previous version with the PA scout, the influence of the scout imaging parameters on the CT tube current was limited to the regions around the skull base and hip joints (Figure 7). In contrast, with the Lat scout or PA + Lat scout, the CT tube current varied substantially depending on the scout imaging parameters. Decreasing the tube voltage or tube current in scout imaging resulted in reductions in CT tube current in the shoulder region and abdominopelvic region. Using the new version, the influence of scout imaging parameters on CT tube current was negligible with the PA scout or PA + Lat scout. With the Lat scout, decreasing the tube voltage or tube current in scout imaging resulted in reduced CT tube current in the abdominopelvic region.

**Figure 8:**
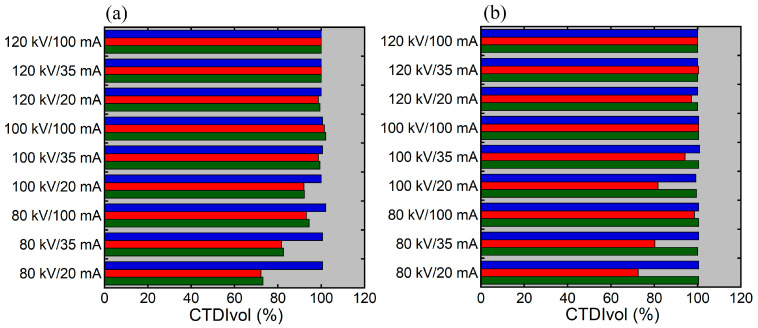
Relative CTDIvol in relation to scout imaging parameters using the previous (**a**) and new (**b**) versions of CARE Dose 4D. The blue, red, and green bars indicate the results with the PA scout, Lat scout, and PA + Lat scout, respectively.

Figure 8 shows the relationship between relative CTDIvol and scout imaging parameters. Regardless of the version of CARE Dose 4D, the influence of the scout imaging parameters on relative CTDIvol was evident with the Lat scout but not with the PA scout. With the PA + Lat scout, the results differed between the previous and the new versions. Relative CTDI varied depending on the scout imaging parameters using the previous version, whereas such variations were not observed using the new version.

**Figure 9:**
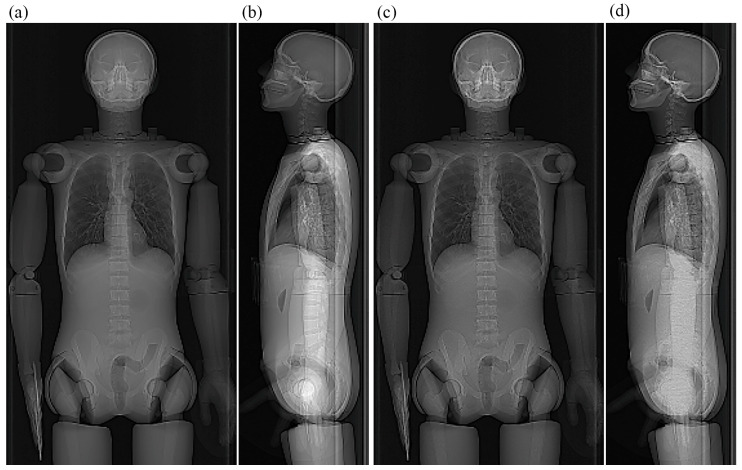
PA (**a**,**c**) and Lat (**b**,**d**) scout images acquired at 120 kV and 100 mA (**a**,**b**) and at 80 kV and 20 mA (**c**,**d**) on the mCT scanner.

When the Lat scout image was acquired at 120 kV and 100 mA, the lumbar vertebrae, which overlapped with the upper extremities, were visible; however, when acquired at 80 kV and 20 mA, the lumbar vertebrae could not be identified (Figure 9). These findings were observed for both scanners. Such loss of contrast was not found on the PA scout images.

## 4. Discussion

The effects of scout direction on the CT tube current determined by AEC were evaluated in imaging the cylindrical phantom and anthropomorphic phantom positioned at the scanner isocenter. When using the previous version of CARE Dose 4D, the CT tube current and thus the radiation dose varied depending on the scout direction, in line with previous reports [7,8,10,11,12,13]. It is noteworthy that the effect of the scout direction varied depending on the imaging object. When the CT dose determined with the PA scout was compared with that with the Lat scout, it was higher in imaging the cylindrical phantom and in imaging the thoraco-abdominopelvic region of the anthropomorphic phantom with the upper extremities raised, except for the mid-thoracic region. However, the CT dose was higher in the head and proximal thighs but lower in the trunk in whole-body imaging of the anthropomorphic phantom with the upper extremities beside the trunk. The CT dose determined with the PA + Lat scout was similar to that with the Lat scout in imaging the cylindrical phantom and in imaging the thoraco-abdominopelvic region of the anthropomorphic phantom with the upper extremities raised. However, it was similar to that with the PA scout from the head to the chest and to that with the Lat scout in the abdominopelvic region.

Using the new version, the effects of the scout direction on the CT dose were relatively small in imaging the cylindrical phantom and the thoraco-abdominopelvic region of the anthropomorphic phantom with the upper extremities raised. In whole-body imaging of the anthropomorphic phantom with the upper extremities beside the trunk, a large discrepancy was found in the lower thoracic region depending on the scout direction, showing a lower dose with the Lat scout than with the PA scout and PA + Lat scout. The effects of the scout direction on the CT dose were confirmed to vary according to the imaging object and were demonstrated to differ between the two versions of CARE Dose 4D. The shape of the dose modulation curve was similar between the PA scout and PA + Lat scout, which is consistent with the software specification that the CT tube current is first determined with the PA scout and then corrected for vertical off-centering based on the Lat scout image.

The influence of vertical positioning on the CT dose determined by AEC was evaluated using the cylindrical phantom and anthropomorphic phantom. When imaging the cylindrical phantom and the thoraco-abdominopelvic region of the anthropomorphic phantom with the upper extremities raised, the CT tube current and thus the radiation dose determined based on the PA scout varied depending on vertical positioning, regardless of the CARE Dose 4D version: a higher dose at lower positions and a lower dose at higher positions. These findings are attributable to the effects of vertical positioning on image magnification, consistent with previous reports [11,14,15,16]. When the Lat scout or PA + Lat scout was used, the effects of vertical positioning on the CT dose were small for both versions. For the previous version, the lower dependence observed with the PA + Lat scout is likely attributable to the similarity in dose modulation between the Lat scout and PA + Lat scout. For the new version, it suggests that the intended off-center correction using the Lat scout image functioned appropriately.

We investigated the influence of scout imaging parameters on the CT dose determined by AEC, using an anthropomorphic phantom. The upper extremities were positioned beside the trunk, and CT was performed from the vertex to the proximal thighs. For both the previous and new versions of CARE Dose 4D, when the CT tube current was determined based on the PA scout, the effect of scout imaging parameters on the CT tube current was minimal, and impacts on CTDIvol were not evident. When the Lat scout was used, decreasing the tube voltage or tube current in scout imaging reduced the CT tube current, particularly in the abdominopelvic region, for both the previous and new versions. Visual assessment of the scout image showed decreased image contrast in the abdominopelvic region on the Lat scout image, which appears to have influenced the dose modulation. These results were in line with the previous study simulating chest CT [8].

With the PA + Lat scout, the scout imaging parameters affected the CT tube current using the previous version, whereas such effects were not observed using the new version. This discrepancy appears to be attributable to the difference in the role of the Lat scout image in dose modulation based on the PA + Lat scout. The Lat image largely affects dose modulation based on the PA + Lat scout when using the previous version, whereas the Lat scout image is used solely for off-center correction when using the new version and the contrast on the Lat image is not important for dose modulation. Decreasing the tube voltage or tube current in scout imaging reduces the patient radiation dose from scout imaging itself and does not cause problems in CT dose modulation when using the new version.

In the experiments assessing the effects of scout imaging parameters, the upper extremities were placed beside the trunk, which increased X-ray attenuation in the Lat direction and is likely to have strengthened the effects on CT dose modulation. On the other hand, although scout imaging parameters did not affect CT dose modulation with the PA scout in this study, decreasing the tube voltage or tube current in scout imaging may impair the contrast of the PA scout image, influencing the CT dose, in a large patient having a long anteroposterior diameter. When using low-dose scout imaging, verifying the contrast on the scout images in each CT examination may be useful to ensure appropriate dose modulation.

This study has some limitations. For optimization of radiological imaging, image quality and diagnostic performance should be considered in addition to the radiation dose. We demonstrated the effects of various factors on CT dose modulation but did not evaluate image quality; therefore, the clinical impact of the differences in CT dose modulation remains unknown. This study showed that the influence of the scout direction on CT dose modulation varied depending on imaging objects. Because patient body habitus is variable, future studies using data from various phantoms and patients are desirable. The influence of scout imaging parameters also remains to be evaluated based on larger phantoms and patients. In this study, we compared two versions of CARE Dose 4D; however, each version was installed in different hardware. Factors other than the software version, such as differences in the imaging table, may have contributed to the observed differences in dose modulation. Studies using different versions installed on the same hardware are desirable. We did not assess the reproducibility of dose modulation using CARE Dose 4D, which remains to be studied.

## 5. Conclusions

The behaviors of two versions of CARE Dose 4D, Siemens AEC software, were investigated. The scout direction influenced CT dose modulation substantially, and the influence differed depending on the imaging object and the software version. The CT tube current determined with the PA scout varied with the vertical positioning of the phantom, presumably due to changes in image magnification; however, such effects were small with the Lat scout or PA + Lat scout. Decreasing the tube voltage or tube current in scout imaging did not affect the CT dose determined with the PA scout, but it influenced the dose determined with the Lat scout. With the PA + Lat scout, the effects of scout imaging parameters on the CT dose were evident using the previous version but minimal using the new version. Off-center correction using the Lat scout image in the new version is suggested to function appropriately and allow a reduction in the scout radiation dose. Because the behavior of AEC is complicated, it is recommended that users examine the characteristics of each AEC system under various imaging conditions.

## Data Availability

The datasets used and analyzed during the current study are available from the corresponding author on reasonable request.

## References

[1] Pearce M.S., Salotti J.A., Little M.P., McHugh K., Lee C., Kim K.P., Howe N.L., Ronckers C.M., Rajaraman P., Craft A.W. (2012). Radiation exposure from CT scans in childhood and subsequent risk of leukaemia and brain tumours: A retrospective cohort study. Lancet.

[2] Meulepas J.M., Ronckers C.M., Smets A.M.J.B., Nievelstein R.A.J., Gradowska P., Lee C., Jahnen A., van Straten M., de Wit M.Y., Zonnenberg B. (2019). Radiation exposure from pediatric CT scans and subsequent cancer risk in The Netherlands. J. Natl. Cancer Inst..

[3] Hauptmann M., Byrnes G., Cardis E., Bernier M.O., Blettner M., Dabin J., Engels H., Istad T.S., Johansen C., Kaijser M. (2023). Brain cancer after radiation exposure from CT examinations of children and young adults: Results from the EPI-CT cohort study. Lancet Oncol..

[4] Smith-Bindman R., Alber S.A., Kwan M.L., Pequeno P., Bolch W.E., Bowles E.J.A., Greenlee R.T., Stout N.K., Weinmann S., Moy L.M. (2025). Medical imaging and pediatric and adolescent hematologic cancer risk. N. Engl. J. Med..

[5] Kalra M.K., Maher M.M., Toth T.L., Schmidt B., Westerman B.L., Morgan H.T., Saini S. (2004). Techniques and applications of automatic tube current modulation for CT. Radiology.

[6] Lee C.H., Goo J.M., Ye H.J., Ye S.J., Park C.M., Chun E.J., Im J.G. (2008). Radiation dose modulation techniques in the multidetector CT era: From basics to practice. Radiographics.

[7] Inoue Y., Nagahara K., Kudo H., Itoh H. (2018). CT dose modulation using automatic exposure control in whole-body PET/CT: Effects of scout imaging direction and arm positioning. Am. J. Nucl. Med. Mol. Imaging.

[8] Franck C., Bacher K. (2016). Influence of localizer and scan direction on the dose-reducing effect of automatic tube current modulation in computed tomography. Radiat. Prot. Dosim..

[9] Iball G.R., Tout D. (2014). Computed tomography automatic exposure control techniques in 18F-FDG oncology PET-CT scanning. Nucl. Med. Commun..

[10] Inoue Y., Nagahara K., Inoki Y., Hara T., Miyatake H. (2019). Clinical evaluation of CT radiation dose in whole-body 18F-FDG PET/CT in relation to scout imaging direction and arm position. Ann. Nucl. Med..

[11] Söderberg M. (2016). OVERVIEW, Practical tips and potential pitfalls of using automatic exposure control in CT: Siemens CARE Dose 4D. Radiat. Prot. Dosim..

[12] Singh S., Petrovic D., Jamnik E., Aran S., Pourjabbar S., Kave M.L., Bradley S.E., Choy G., Kalra M.K. (2014). Effect of localizer radiograph on radiation dose associated with automatic exposure control: Human cadaver and patient study. J. Comput. Assist. Tomogr..

[13] Papadakis A.E., Perisinakis K., Damilakis J. (2008). Automatic exposure control in pediatric and adult multidetector CT examinations: A phantom study on dose reduction and image quality. Med. Phys..

[14] Lambert J.W., Kumar S., Chen J.S., Wang Z.J., Gould R.G., Yeh B.M. (2015). Investigating the CT localizer radiograph: Acquisition parameters, patient centring and their combined influence on radiation dose. Br. J. Radiol..

[15] Paolicchi F., Bastiani L., Negri J., Caramella D. (2020). Effect of CT localizer radiographs on radiation dose associated with automatic tube current modulation: A multivendor study. Curr. Probl. Diagn. Radiol..

[16] Kaasalainen T., Palmu K., Reijonen V., Kortesniemi M. (2014). Effect of patient centering on patient dose and image noise in chest CT. AJR Am. J. Roentgenol..

[17] Martí-Climent J.M., Prieto E., Morán V., Sancho L., Rodríguez-Fraile M., Arbizu J., García-Velloso M.J., Richter J.A. (2017). Effective dose estimation for oncological and neurological PET/CT procedures. EJNMMI Res..

[18] Inoue Y., Itoh H. (2020). Effects of scout radiographic imaging conditions on tube current modulation in chest computed tomography. J. Radiol. Prot..

[19] Schmidt B.T., Hupfer M., Saltybaeva N., Kolditz D., Kalender W.A. (2017). Dose optimization for computed tomography localizer radiographs for low-dose lung computed tomography examinations. Invest. Radiol..

[20] Hadi Y.H., Keaney L., England A., Moore N., McEntee M. (2025). Automatic patient centering in computed tomography: A systematic review and met a-analysis. Eur. Radiol..

